# “*PrEP Gives the Woman the Control”:* Healthcare Worker Perspectives on Using pre-Exposure Prophylaxis (PrEP) During Pregnancy and Postpartum in Kenya

**DOI:** 10.1177/23259582221111068

**Published:** 2022-07-01

**Authors:** Nancy Mwongeli, Anjuli D. Wagner, Julia C Dettinger, Jillian Pintye, Susan Brown Trinidad, Merceline Awuor, Grace Kimemia, Kenneth Ngure, Renee A. Heffron, Jared M. Baeten, Nelly Mugo, Elizabeth A. Bukusi, John Kinuthia, Maureen C. Kelley, Grace C. John-Stewart, Kristin M. Beima-sofie

**Affiliations:** 1 285569 Kenyatta National Hospital, Nairobi, Kenya; 2 Department of Global Health, 7284 University of Washington, Seattle, WA, USA; 3 School of Nursing, 7284 University of Washington, Seattle, WA, USA; 4 Department of Bioethics and Humanities, 7284 University of Washington, Seattle, WA, USA; 5 7284 University of Washington Kenya (UW-Kenya), Nairobi, Kenya; 6 Population Dynamic and Reproductive Health, 107883 African Population and Health Research Center, Nairobi, Kenya; 7 Department of Community Health, 118985 Jomo Kenyatta University of Agriculture and Technology, Juja, Kenya; 8 Department of Epidemiology, 7284 University of Washington, Seattle, WA, USA; 9 Department of Medicine, 7284 University of Washington, Seattle, WA, USA; 10 2158 Gilead Sciences, Foster City, USA; 11 Partners in Health Research and Development, Thika, Kenya; 12 185955 Centre for Clinical Research, Kenya Medical Research Institute (KEMRI), Nairobi, Kenya; 13 Department of Obstetrics and Gynecology, 7284 University of Washington, Seattle, WA, USA; 14 The Ethox Centre and 575097 Wellcome Centre for Ethics & Humanities, Nuffield Department of Population Health, University of Oxford, Oxford, UK; 15 Department of Pediatrics, 7284 University of Washington, Seattle, WA, USA

**Keywords:** pre-exposure prophylaxis (PrEP), HIV prevention, pregnancy, postpartum, healthcare workers

## Abstract

**Background:** Pregnant and postpartum women in high HIV prevalent regions are at increased HIV risk. Oral pre-exposure prophylaxis (PrEP) can decrease HIV incidence reducing infant HIV infections. Understanding healthcare worker (HCW) beliefs about PrEP prior to national roll-out is critical to supporting PrEP scale-up. **Methods:** We conducted 45 semi-structured interviews among a range of HCW cadres with and without PrEP provision experience purposively recruited from four clinics in Kenya to compare their views on prescribing PrEP during pregnancy and postpartum. Interviews were analysed using a conventional content analysis approach to identify key influences on PrEP acceptability and feasibility. **Results:** All HCWs perceived PrEP as an acceptable and feasible HIV prevention strategy for pregnant and postpartum women. They believed PrEP meets women’s needs as an on-demand, female-controlled prevention strategy that empowers women to take control of their HIV risk. HCWs highlighted their role in PrEP delivery success while acknowledging how their knowledge gaps, concerns and perceived PrEP implementation challenges may hinder optimal PrEP delivery. **Conclusion:** HCWs supported PrEP provision to pregnant and postpartum women. However, counseling tools to address risk perceptions in this population and strategies to reduce HCW knowledge gaps, concerns and perceived implementation barriers are required.

## Introduction

Women are at an elevated risk of acquiring HIV during pregnancy and postpartum.^1,2^ Incident HIV infection during this crucial period is often unrecognized and untreated^3^ resulting in higher rates of vertical transmission among women with incident infections compared to women with chronic HIV infection.^4,5^ Postpartum HIV acquisition contributes to an increasing proportion of infant HIV infections and challenges attainment of elimination of mother-to-child-transmission (eMTCT) goals.^6^

Kenya is a high HIV burden country with a generalized epidemic driven primarily by heterosexual transmission.^7^ Barriers to utilizing existing HIV combination prevention strategies among pregnant and postpartum women include low awareness of partners’ HIV status and difficulty negotiating mutual monogamy or condom use.^8^ Oral pre-exposure prophylaxis (PrEP) serves as a promising and discreet HIV prevention strategy for women,^9^ who currently have limited prevention options, and can contribute to eMTCT by addressing Prong 1 in the World Health Organization (WHO) prevention of mother-to-child transmission (PMTCT) strategy: primary HIV prevention in women.^10^

WHO recommends offering PrEP to people at substantial risk of HIV acquisition as part of combination HIV prevention services.^11^ Oral PrEP is highly effective with high adherence, and is safe during pregnancy and breastfeeding.^12^ Healthcare workers (HCWs) are the gatekeepers for this intervention as they are responsible for identifying women who could benefit from PrEP, counseling women on its use, assisting women in making informed choices on whether to initiate PrEP and following up PrEP users to ensure persistence and adherence.^13^

Therefore, understanding HCWs’ perceptions, concerns and considerations while prescribing PrEP during pregnancy and postpartum is critical in understanding how to optimize PrEP delivery and ensure high efficiency and maximal impact. Understanding the perspectives of early adopting HCWs can inform the development of policies regarding larger scale implementation in routine clinical care,^14^ identify important considerations for other regions or settings that are now considering PrEP provision in pregnancy and postpartum and inform strategies of implementing other novel PrEP agents in the pipeline. This study aimed to identify the acceptability and feasibility of PrEP implementation among Kenyan HCWs offering services to pregnant and postpartum women.

## Methods

### Study Design and Population

Between July 2015 and January 2016, we conducted 45 semi-structured interviews with PrEP-experienced and PrEP-naïve HCWs providing care to pregnant women attending 4 clinics in high HIV prevalence regions in Kenya. Clinics selected had an antenatal (ANC) HIV prevalence that was higher than the national average (>8.3%) and large numbers of pregnant and postpartum women seeking care.^15,16^ Sites included an ethnically diverse population. PrEP-naïve HCWs were recruited from 2 sites: Mathare North Health Centre, an urban site within Nairobi, and Ahero County Hospital, a peri-urban site near Lake Victoria. PrEP-experienced HCWs were recruited from 2 sites participating in the Partners Demonstration Project (an open-label implementation project evaluating integrated delivery of PrEP and antiretroviral therapy in Thika and Kisumu that had been providing PrEP since 2012)^17,18^ Thika level 5 Hospital, an urban site near Nairobi, and Kisumu County Hospital, an urban site in Kisumu county.

### Recruitment and Data Collection

Within each clinic, HCWs including clinical officers (HCWs with a clinical medicine diploma/degree), nurses (HCWs completed formal nursing education), nurse counselors, mentor mothers, pharmacists, community health workers and psychologists were purposively recruited. Both clinical officers and nurses offer primary care and can prescribe PrEP. Interviews were conducted prior to national PrEP roll-out.^19^ PrEP naïve HCWs were defined as HCWs who had never been trained on, counseled, or prescribed PrEP. PrEP experienced HCWs had up to 3 years of experience providing PrEP through involvement in the Partners Demonstration Project. PrEP naïve HCWs were included in order to understand whether experience with PrEP influenced provider attitudes.

Semi-structured interview guides were developed collaboratively by study team members (KBS, SBT, KN, and MK) based on literature reviews and experiences in research ethics and HIV prevention research. Interview guides were piloted with Kenyan investigators, female staff, and HCWs from a neighboring facility, and revised accordingly to ensure clarity and cultural appropriateness. Categories included in the interview guides centered on 3 main topic areas: 1) clinical decision-making regarding medication use during pregnancy and postpartum, 2) the ethical conduct of intervention research during pregnancy and postpartum, and 3) issues specific to the clinical implementation of PrEP during pregnancy and postpartum (Supplementary file 1). HCWs were informed about the study in-person by study staff and HCWs interested in participating were referred to trained, female, social scientist interviewers with no prior relationship with participants (KBS, SBT, MG, LA, and MA) to conduct study interviews. All HCWs participated in a single interview that was conducted in English in a private room at the clinic, was audio recorded, and transcribed verbatim. Interviews ranged between 33 and 112 minutes in length. Detailed field notes were written for each interview.

### Data Analysis

The primary goal of this analysis was to identify HCW beliefs about the acceptability and feasibility of providing PrEP to pregnant and postpartum women. We conducted a conventional content analysis to produce the key concepts and themes related to the clinical implementation of PrEP during pregnancy and postpartum periods.^20^ A codebook was developed iteratively by the primary analysis team (KBS, SBT, JP, MA, GK) through a process of reading and re-reading transcripts, code development and refinement, code application to transcripts, discussion of code application, and revision of codes and code definitions. The final codebook included codes to capture: 1) Decision making factors, 2) Decision making context for PrEP use, 3) Comparisons for PrEP use, 4) Decision making roles, 5) PrEP use, 6) Implementation facilitators and barriers, and 7) PrEP use and partnership, culture, gender norms and community. The primary analysis team consisted of a combination of Kenyan and US-based women with considerable experience in HIV prevention and analyzing qualitative data. Following code book development, the primary analysis team participated in consensus coding to ensure agreement on code application across team members. Transcripts and codes were imported into ATLAS.ti v.7 (Scientific Software Development GmbH, Berlin, Germany) for data management and analysis. All transcripts were coded independently by one member of the study team (KBS, SBT, JP, GK) using the final version of the codebook then reviewed by another team member (NM, KBS, SBT, JP, GK). Disagreements in code application were resolved through group discussions. Queries were run to identify convergent and divergent themes within and between PrEP-naïve and PrEP-experienced HCWs.^21^ Conventional content analysis techniques were used to map convergent and divergent themes within and between population groups and code categories.^22^ Transcripts and study results were not shared with participants for validation; however, interviewers were included on the analysis team to ensure findings reflected participant experiences.

### Ethical Approval and Informed Consent

The study was approved by the Kenya Medical Research Institute Ethical Review Committee (ERC) (approvals: 3080 and 3083), the Kenyatta National Hospital/University of Nairobi ERC (approval: P98/02/2015), and the University of Washington Institutional Review Board (approvals: 48911 and 49168). All participants provided written informed consent prior to study enrollment

## Results

We conducted a total of 45 interviews with HCWs, including 25 with experience providing PrEP and 20 without PrEP provision experience. HCWs represented a range of cadres, including nurses (n = 7), nurse counselors (n = 8), clinical officers (n = 7), and counselors (n = 5), among others. HCWs were a median age of 36 (IQR: 31-39) and reported a median of 8 years (IQR: 4-10) of experience working with pregnant women. The majority of HCWs were female (67%) and represented diverse ethnic backgrounds. Fewer PrEP experienced HCW were women, they represented slightly different ethnicity groups, and had a different distribution of cadres as the PrEP-naïve HCW (Table 1).

**Table 1.** Healthcare Worker Demographic Characteristics.

Characteristic	Total	PrEP experienced	PrEP naïve
	**n (%) or Median (IQR)**		
Age	36 (31-39)	36 (32-38)	35.5 (28.5, 40.5)
Female	30 (67)	12 (48)	18 (90)
Has children	34 (76)	17 (68)	17 (85)
Ethnicity			
Embu	1 (2)	1 (4)	--
Kikuyu	12 (27)	8 (32)	4 (20)
Kisii	5 (11)	2 (8)	3 (15)
Luhya	4 (9)	3 (12)	1 (5)
Luo	17 (38)	10 (40)	7 (35)
Meru	2 (4)	1 (4)	1 (5)
Kamba	4 (9)	--	4 (20)
Clinical training			
Nurse	7 (16)	1 (4)	6 (30)
Nurse Counselor	8 (18)	4 (16)	4 (20)
Counselor	5 (11)	4 (16)	1 (5)
Clinician	1 (2)	--	1 (5)
Clinical Officer	7 (16)	5 (20)	2 (10)
Community health worker	4 (9)	4 (16)	--
Nurse psychologist	1 (2)	1 (4)	--
Pharmacist	4 (9)	4 (16)	--
Psychologist	2 (4)	2 (8)	--
Peer counselor	4 (9)	--	4 (20)
Other	2 (4)	--	2 (10)
Years of experience (total)	9 (6-13)	10 (8, 11)	7.5 (5, 15)
Years working with pregnant women	8 (4-10)	8 (6, 10)	6 (3.5, 10)

Three major themes emerged from the data, reflecting the main opinions and concerns of HCWs related to oral PrEP provision among pregnant and postpartum women: 1) Oral PrEP is an acceptable HIV prevention strategy and meets the needs of pregnant and postpartum women, 2) HCW knowledge gaps in eligibility and risk assessment may limit HCW implementation, 3) Multiple facility and interpersonal level barriers may limit the feasibility of oral PrEP implementation.

### Oral PrEP is an Acceptable HIV Prevention Strategy and Meets the Needs of Pregnant and Postpartum Women

Despite differences in personal experience with PrEP provision, all HCWs expressed support for oral PrEP as an acceptable intervention to reduce HIV incidence among pregnant and postpartum women. Both categories of HCWs expressed no reservations towards prescribing PrEP to pregnant and postpartum women, citing that PrEP is safe in pregnancy and can avert new HIV infections in this population.

“Again PrEP, because it is safe for pregnancy, I think it is even now better because it does not mean that when a lady becomes pregnant she stops having sex, she will continue having sex and she still needs the protection… as for me as a person, I would not have any challenges with a lady using PrEP because still they are at high risks of getting infected giving that they are [living in] a discordant relationship.” - PrEP-experienced Counsellor

Several HCWs believed that PrEP could overcome gender disparities that hinder uptake of HIV prevention strategies, offering hope to pregnant and postpartum women with limited personal prevention options, including women facing difficulties negotiating condom use or are unaware of their partner’s HIV status. HCWs felt that women would be able to discreetly take PrEP, which could empower women to take control of their health, and ensure they remain HIV-free.

“… maybe this mother has been desperate, that what intervention do I use, to take initiative of the … the woman is in charge now; she is in charge because she is the one using the medication. So even if the man says he does not want to use protection, you don’t have to argue about it, you are safe.” – PrEP-experienced clinical officer

In the setting of a patriarchal society, HCWs also felt that PrEP had a relative advantage over other HIV prevention interventions such as condoms, which require consent/participation from male partners.

“No, I must say [PrEP and condoms] are different because condoms, this is something that you want to talk to somebody else to use, it’s not you using it. But you know PrEP; you are going to give it to the pregnant mother herself so she is sure she is protected. You give a condom, she goes home, the man refuses to use [the] condom, she has no choice. PrEP gives the woman the control” – PrEP-naïve nurse counsellor

HCWs with and without experience prescribing PrEP believed that the demand and the need for PrEP in this population was high, and that pregnant and postpartum women would easily accept PrEP. HCWs who had prescribed PrEP during the Partners Demonstration Project noted how participants became concerned towards the end of the study when they became aware that access to PrEP might end.

“Some [study participants] went and kept some drugs [PrEP] because they know they will be stopped after 2–3 months… they are stocking them for future use when they are stopped… One thing I know is that the need for PrEP is high, that it should be made available.” – PrEP-experienced pharmacist

“[I]f you became pregnant, you can choose to continue with [PrEP] but you come for monthly visits or maybe you can stop but continue with monthly visits or you stop [PrEP] and come after every three months so we used to tell them and they were the ones to choose, and most of them wanted to continue with [PrEP].” – PrEP-experienced nurse counsellor

HCWs felt that the woman’s risk perception drives PrEP acceptability, use, and adherence. However, several HCWs noted that there could be misalignment between a woman’s perceived risk and her actual risk, posing a challenge for PrEP counseling. HCWs felt that pregnant and postpartum women who were aware of their own risk were easier to counsel, and more likely to initiate, use, and adhere to PrEP, compared with those who believed that they were not at risk.

“The perception of the woman … Do they feel they are at risk? What kind of relationship do they have with their partner and especially about HIV because you see that perception is what will drive the appearance and what [will] drive their commitment to use of PrEP.” – PrEP-experienced community health worker

“[W]hen you counsel somebody who is not sick, it’s difficult. Like you are telling me you want to prevent and but am not sick so I ask, ‘Why do you want to give me medication and am not sick? But when I am sick, you counsel me, I am desperate to get that care’.” – PrEP-experienced nurse counsellor

### Knowledge Gaps Regarding Eligibility and Risk Assessment and HCW Attitudes Might Limit PrEP Implementation

Among HCWs, there was consensus that PrEP implementation is highly dependent on the knowledge and attitudes that HCWs have towards PrEP. Although HCWs expressed no reservations in prescribing PrEP in this population, HCWs with and without experience delivering PrEP described ambiguity in the definition of risk and lack of clarity on PrEP eligibility. Aligned with their personal involvement in the Partners Demonstration Project, or familiarity with other local PrEP trials, HCWs in this study advocated that PrEP should always be offered to women in HIV serodiscordant relationships. However, outside of HIV discordance, the definition of HIV risk and criteria for PrEP eligibility was less clear. Some HCWs believed that all women should be told about PrEP and offered PrEP, while others believed it should be restricted to sero-discordant couples. Many HCWs were uncertain about how PrEP eligibility should be assessed among pregnant and postpartum women.

“[I]f PrEP can prevent HIV during pregnancy, so the woman is negative and she is pregnant and she takes this drug and she doesn’t get HIV, we should then just offer to all women who are pregnant and HIV negative, does that seem… what do you think of that idea?” – PrEP-naïve mentor mother

“[I]t is important that if [PrEP] is taken to [clinics], then the group that takes it must be proven to be discordant couples, instead of being taken by everyone.” – PrEP-experienced community health worker

HCWs who were part of the Partners Demonstration Project described how some women participating in the study would seek ANC care at other facilities for minor health concerns, where they would be persuaded to stop PrEP use by HCWs with less PrEP experience or knowledge.

“There were such where you go to a clinic and you have a minor complication, they will tell you that the condition is due to this [PrEP], stop. Some did stop and when they come here they come with bottles full of drugs then say they were told to stop at ANC clinic.” – PrEP-experienced community health worker

Moreover, HCWs felt that their attitudes towards or against PrEP would determine the information that a pregnant or postpartum woman would be given concerning PrEP, which in turn would greatly influence her uptake, usage, and adherence.

“First the healthcare worker herself, the attitude of the healthcare worker, if I think it’s not a good idea, then it means I will talk less about it.” – PrEP-naïve nurse counsellor

### Multiple Facility and Interpersonal Level Barriers may Limit the Feasibility of Oral PrEP Implementation

As much as HCWs felt that PrEP was a good intervention for pregnant and postpartum women, they felt that its implementation would have several challenges. They raised concerns about increased workload in an already overburdened clinical setting, low adherence to PrEP medication, partner reluctance, and stigma.

HCWs felt that implementing PrEP would increase the workload in already thinly stretched healthcare facilities. They believed that PrEP implementation would be complex, and would require HCWs to spend significant time counseling potential and ongoing PrEP users on the drug itself, risks, and adherence.

“Secondly it is going to add the workload (laughing), that is going to be crazy because right now if you get a HIV positive mother, you take so much time with them, so it means every mother who comes to the clinic, you are going to take more time with them, because you are going to explain to them about PrEP and all that.” – PrEP-naïve nurse counsellor

When considering currently available PrEP options, HCWs felt that taking a pill daily would be a huge burden for pregnant and postpartum women and they may experience challenges with PrEP adherence, therefore reducing its effectiveness. HCWs suggested that other modes of PrEP delivery could overcome this challenge and improve adherence.

“Adherence is the issue, if we can get something like a patch, you know a patch, put it [once] a week, it would be good.” – PrEP-experienced pharmacist

HCWs felt that women’s partners played a major role in the acceptability, use, and adherence to PrEP. They felt that most women would follow their partners’ directives regardless of their own perceived or actual risk, presenting a challenge to PrEP implementation.

“Like a woman who tell you she want to take PrEP but wishes the husband would be there, she would have taken it.” – PrEP-experienced pharmacist

HCWs also believed that community stigma could prevent PrEP uptake among women who want to use PrEP.

“So there is that kind of…should I call it a stigma thing, people don’t want to associate with you because of the drugs. So this mother will feel, “Why should I carry this bunch of drug?” Even though they are for my prevention, so that acceptance is not there.” – PrEP-naïve nurse

## Discussion

HCWs believed that oral PrEP is a highly promising HIV prevention intervention for pregnant and postpartum women who desire to protect themselves and their infants from HIV, but who may have limited control over their own HIV risk. These beliefs did not differ between HCWs with and without experience providing PrEP, suggesting the potential of PrEP as a promising HIV prevention method for pregnant and postpartum women was clear even to HCWs without any previous PrEP training or experience. HCWs found PrEP to be preferable to condoms for HIV prevention for pregnant and postpartum women living in a patriarchal society, portraying the two as alternatives, rather than complementary combination strategies, as recommended in the WHO HIV prevention guidelines.^23^ HCWs felt that they played a major role in delivering PrEP to women, and that HCW knowledge, attitudes and concerns were determinants of successful implementation of PrEP in this population. Finally, HCWs foresaw several challenges in PrEP implementation related to partner reluctance, community stigma, and increased HCW workload.

Responses from HCWs highlighted their fear of misalignment between the women’s HIV risk perception and empiric HIV risk. They were concerned that pregnant and postpartum women might underestimate their risk of infection, and thus not take advantage of PrEP. In a Kenyan study among adolescent girls and young women (AGYW), 43% of those with at least one risk factor for HIV acquisition who did not initiate PrEP did so because they did not perceive themselves to be at risk.^24^ Among those who did initiate PrEP, low risk perception was the most common reason for discontinuation. HIV risk perception was noted to be low even among women who did not know their partners’ HIV status.^24^ However, in a separate study in Kenya among pregnant and postpartum women offered PrEP, an empiric risk score developed for the pregnancy period^25^ was compared to several scores measuring self-perceived risk. Women with higher empiric risk reported significantly higher self-perceived risk, suggesting that pregnant women may accurately assess their own risk.^26^ While risk assessment tools are being used by the Kenyan government for PrEP delivery and by research studies^27^ to provide risk-guided PrEP counseling and services to pregnant women, it is not yet clear whether these tools modify women’s risk perception or PrEP decision-making.

HCWs reported some knowledge gaps and revealed concerns in their perceptions about PrEP’s impact on women’s behavior. HCWs lacked clarity around PrEP eligibility beyond serodiscordant couples. This may be attributable to this study collecting data early during PrEP implementation in Kenya and sampling a number of HCWs who had experience delivering PrEP to women within sero-discordancy clinics and studies. Additionally, while HCWs reported believing that PrEP is safe and should be provided to pregnant and postpartum women, knowledge gaps and biases might interfere with PrEP counseling and prescription. To aid in PrEP scale up and address these knowledge gaps and biases, training and mentorship of HCWs, development of standardized PrEP curricula and job aides, paired with interventions like values clarification activities and standardized patient actor trainings, could be utilized to overcome HCW bias and knowledge gaps.^28,29^

HCWs identified challenges in PrEP implementation related to increased HCW workload, poor adherence, partner reluctance, and community stigma. These findings align with other studies conducted among HCW providing care to AGYW, and pregnant women in Kenya, South Africa, Tanzania, and Zimbabwe. Studies conducted among HCW providing care to AGYW found that while providers were accepting of PrEP as an option, they reported significant concerns around community stigma, family and partner dynamics, concerns about encouraging sexual behavior among adolescent girls in particular, and adherence challenges in this population.^30,31^ A study of South African HCW providing care to pregnant women found that provider knowledge about PrEP, particularly in the context of pregnancy, was low overall and among those who reported knowing about PrEP there remained significant gaps in knowledge that would impact appropriate PrEP delivery to pregnant women.^32^ A broader implementation science review found similar challenges to implementation of PrEP delivery to pregnant and postpartum women.^33^ The review highlighted ongoing studies testing implementation strategies to overcome these barriers, such as task-shifting of PrEP counseling, integration of PrEP services in maternal and child health clinics, community education to improve PrEP awareness, and self-assessment of HIV risk during antenatal and postnatal clinics.^33^ Identifying strategies that optimize PrEP delivery specifically for pregnant and postpartum women will be essential to broaden scale up globally.

**Limitations:** HCW interviews were conducted before the national roll-out of PrEP in Kenya. At this time, HCWs were most familiar with PrEP clinical trials and demonstration projects among sero-discordant couples; therefore, their views may not be generalizable to all PrEP providers in MCH, nor those delivering services currently.

## Conclusion

HCWs perceive PrEP as an acceptable, feasible, and empowering HIV prevention strategy among pregnant and postpartum women in Kenya and would be willing to prescribe it. They observed demand and need of PrEP but noted that proper strategies and tools are required to counsel the women on their HIV risk to ensure maximal and efficient utilization of PrEP in this population. HCWs suggested that training to address both clinical knowledge gaps and PrEP-related stigma, job aides on PrEP delivery and efficient implementation strategies to overcome the barriers of PrEP delivery perceived by HCWs could help ensure maximal delivery of PrEP to pregnant and postpartum women.

## Supplemental Material

sj-pdf-1-jia-10.1177_23259582221111068 - Supplemental material for “*PrEP Gives the Woman the Control”:* Healthcare Worker Perspectives on Using pre-Exposure Prophylaxis (PrEP) During Pregnancy and Postpartum in Kenya Click here for additional data file. Supplemental material, sj-pdf-1-jia-10.1177_23259582221111068 for “*PrEP Gives the Woman the Control”:* Healthcare Worker Perspectives on Using pre-Exposure Prophylaxis (PrEP) During Pregnancy and Postpartum in Kenya by Nancy Mwongeli, Anjuli D. Wagner, Julia C Dettinger, Jillian Pintye, Susan Brown Trinidad, Merceline Awuor, Grace Kimemia, Kenneth Ngure, Renee A. Heffron, Jared M. Baeten, Nelly Mugo, Elizabeth A. Bukusi, John Kinuthia, Maureen C. Kelley, Grace C. John-Stewart and Kristin M. Beima-sofie in Journal of the International Association of Providers of AIDS Care (JIAPAC)
